# Supplementary material for: Causal interplay between lactose intolerance and gut microbiota: a combined bidirectional Mendelian randomization and in vivo validation study
Source: Front Nutr. 2026 Jun 1;13:1803337. doi: 10.3389/fnut.2026.1803337 (PMC13265576; doi:10.3389/fnut.2026.1803337)
Supplement: Supplementary file 1 [file Data_Sheet_1.zip › supplementary materials/Forward/leave one out cross validation/ebi-a-GCST90027828.finngen_R12_E4_LACTONAS.pdf]

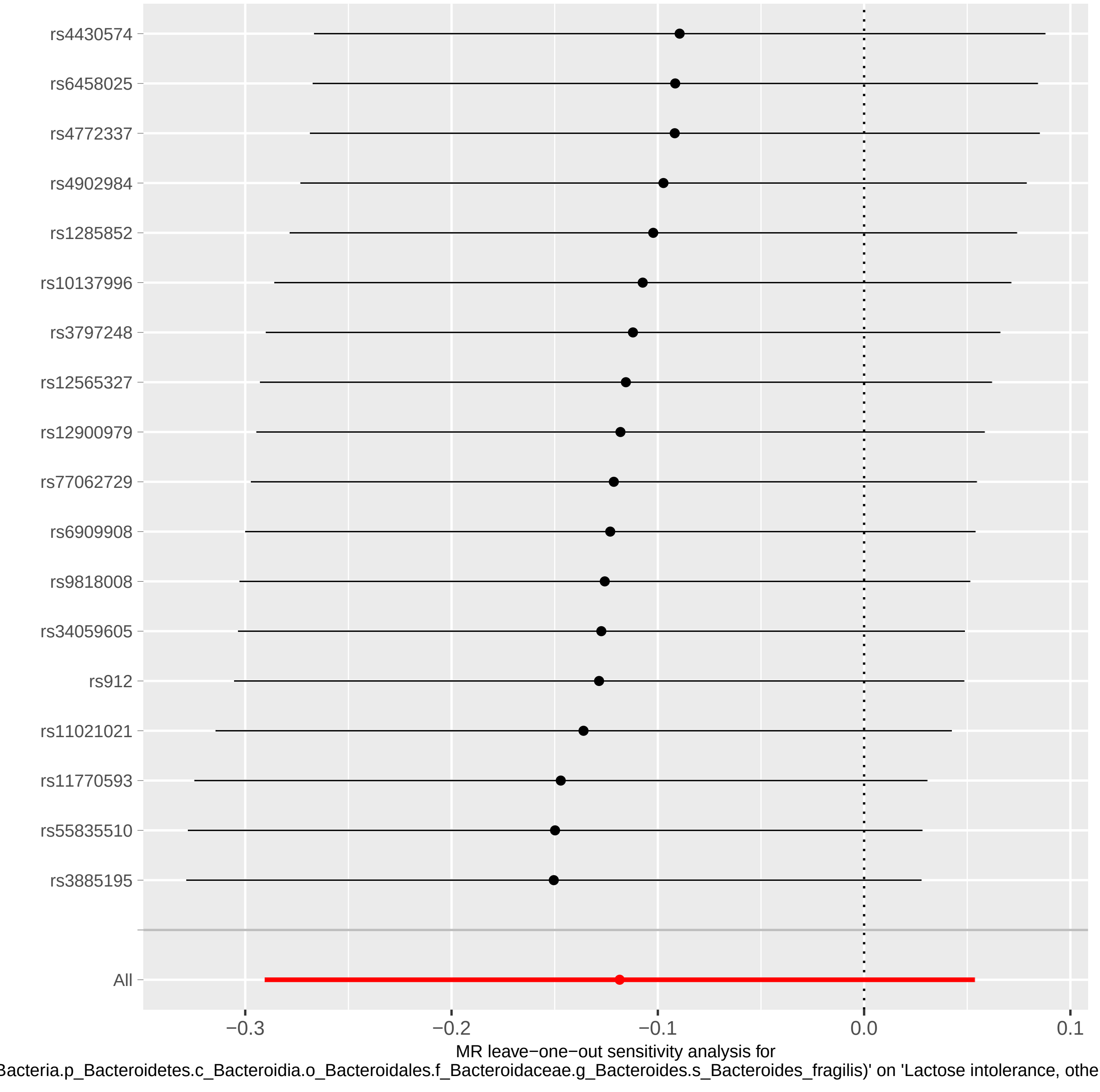
rs4430574
rs6458025
rs4772337
rs4902984
rs1285852
rs10137996
rs3797248
rs12565327
rs12900979
rs77062729
rs6909908
rs9818008
rs34059605
rs912
rs11021021
rs11770593
rs55835510
rs3885195
All
−0.3
−0.2
−0.1
0.0
0.1
MR leave−one−out sensitivity analysis for
Bacteria.p_Bacteroidetes.c_Bacteroidia.o_Bacteroidales.f_Bacteroidaceae.g_Bacteroides.s_Bacteroides_fragilis)' on 'Lactose intolerance, othe
